# Candida tropicalis Fungal Keratitis: A Case Report and Literature Review

**DOI:** 10.7759/cureus.62682

**Published:** 2024-06-19

**Authors:** Soukaina Adadi, Khadija Jarnige, Rabi Issaka Amidou, Youssef Kfal, Zineb Tlamcani

**Affiliations:** 1 Parasitology and Mycology Department, Central Laboratory of Medical Analyses, Hassan II University Hospital Center/Faculty of Medicine, Pharmacy and Dentistry, Sidi Med Ben Abdellah University, Fez, MAR

**Keywords:** mycological study, infection, corneal abscess, candida tropicalis, fungal keratitis

## Abstract

Fungal keratitis, or keratomycosis, is an infection of the cornea caused by fungi. Although it is less frequently implicated in ocular infections than bacterial keratitis, its prognosis remains more guarded. However, the fungi involved include a variety of rare fungal species. Fungal keratitis caused by *C. tropicalis *has been reported only rarely in the literature.

We report the first case of *Candida tropicalis *corneal abscess diagnosed in the Parasitology-Mycology Department of the Hassan II University Hospital in Fez: a 66-year-old patient with corneal dystrophy was admitted to the Ophthalmology Department for management of a corneal abscess of the left eye. Fungal infection was confirmed by mycological study of the corneal scrapings. The patient was put on antifungal treatment with good clinical improvement.

## Introduction

Fungal keratitis, also known as keratomycosis, is among the most serious types of corneal infections, primarily due to its diagnostic and treatment complexities [1]. Keratomycosis is characterized by an invasive infection of the corneal stroma caused by specific, opportunistic fungal pathogens [1]. It is regarded as a rare condition, although a slight increase in its prevalence has been reported [1]. Various agents responsible for fungal keratitis have been reported, with *Candida albicans* long recognized as a cause of fungal keratitis [2]. In recent years, new species of the *Candida* genus have also been identified as a cause of ocular infection, including *Candida tropicalis* as a causative agent of keratomycosis. This case report aims to describe a case of *Candida tropicalis* infectious keratitis, the first case reported in Morocco to our knowledge.

## Case presentation

The patient, 66 years old, has been followed for 30 years for corneal dystrophy, characterized by a progressive decrease in visual acuity with recurrent ocular pain. Five days before admission to the ophthalmology department, he presented with acute pain and redness in the left eye, accompanied by decreased visual acuity, with no history of trauma, self-medication, or application of traditional treatments. The patient also has a family history of corneal dystrophy, affecting his father and four of his children.

Ophthalmological examination showed that the eyelids are correctly positioned, have a normal shape, and function effectively with slight palpebral edema, the presence of purulent secretions, slight chemosis inferiorly, and an opaque dystrophic cornea with 360° vascular appeal. Fluorescein examination showed a 7*5 mm superior nasal ulcer.

The diagnosis of pearly-white epitheliostromal subtotal corneal abscess was retained, and the decision was made to hospitalize the patient, take corneal and conjunctival samples for bacteriological and mycological study, then put the patient on local and general antibiotic treatment to treat the superinfection.

Mycological examination 

The corneal swab received was suspended in a small amount of saline solution, vortexed, and then utilized for mycological study, including direct examination and culture. The initial direct examination yielded negative results.

The culture was performed on Sabouraud simple, Sabouraud chloramphenicol (SC), and Sabouraud actidione (SA) media. Incubation was carried out in the oven at 37°C and 27°C.

**Figure 1:**
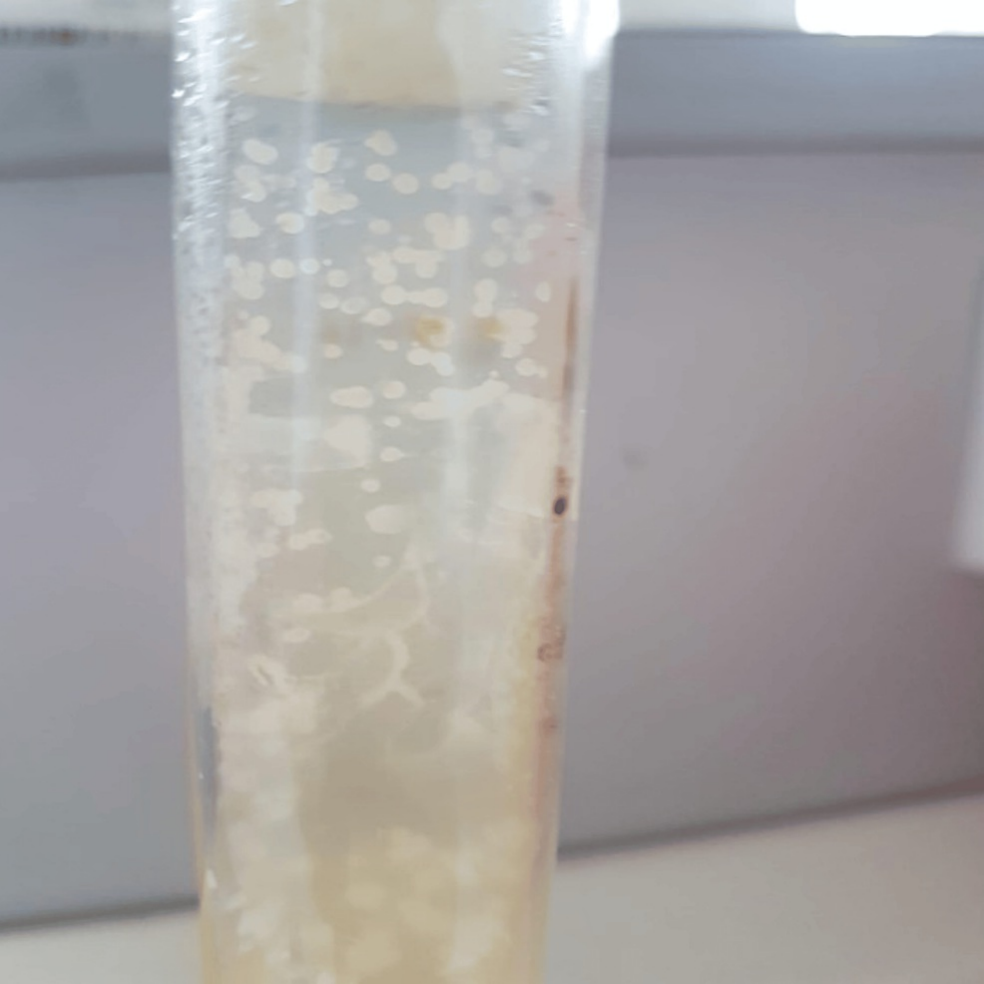
Macroscopic appearance of colonies on Sabouraud chloramphenicol medium (Photo courtesy of the Parasitology-Mycology Department, CHU Hassan II, Fez).

Two days later, creamy, smooth, whitish colonies appeared on all three media (Figure 1).

**Figure 2:**
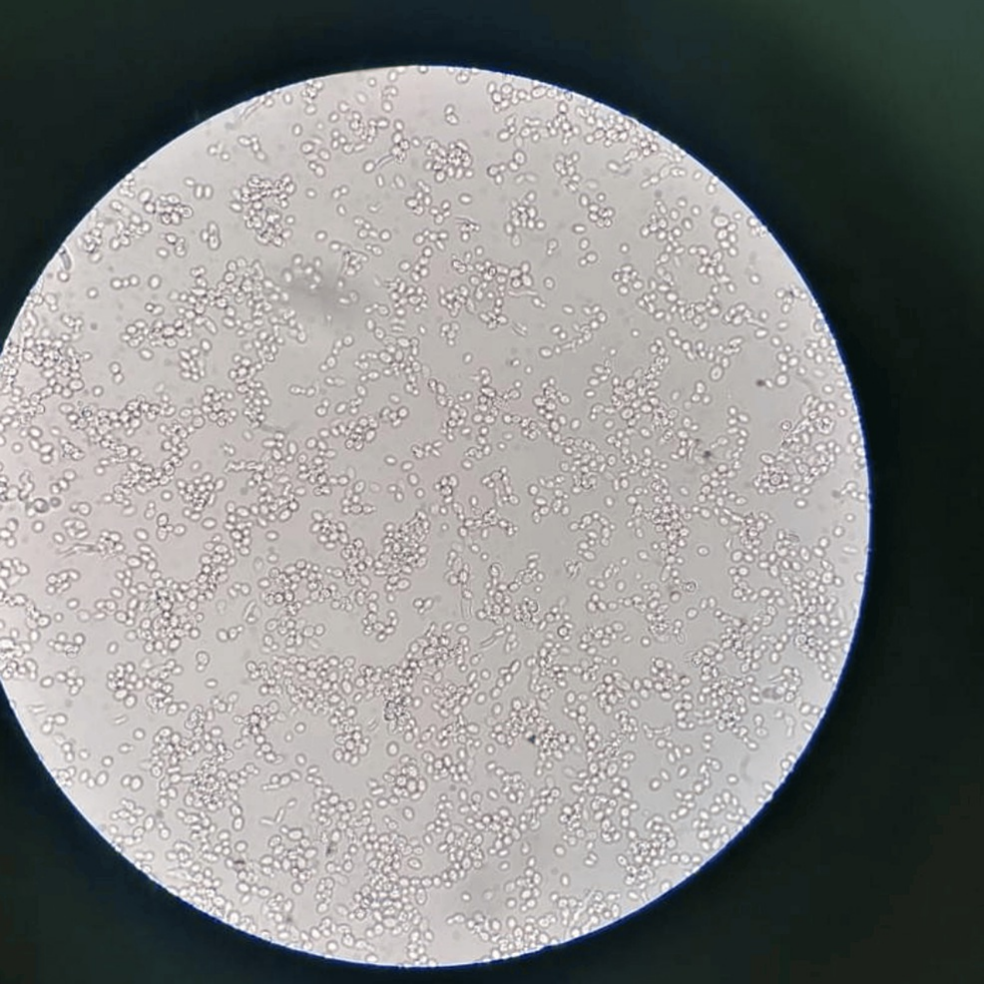
Examination of colonies between slide and coverslip shows the presence of yeasts, magnification X 400 (photo by the Parasitology-Mycology Department, CHU Hassan II, Fez.

A direct slide-to-slide examination was carried out on the culture, showing the presence of thin-walled yeasts with multilateral budding (Figure 2).

**Figure 3:**
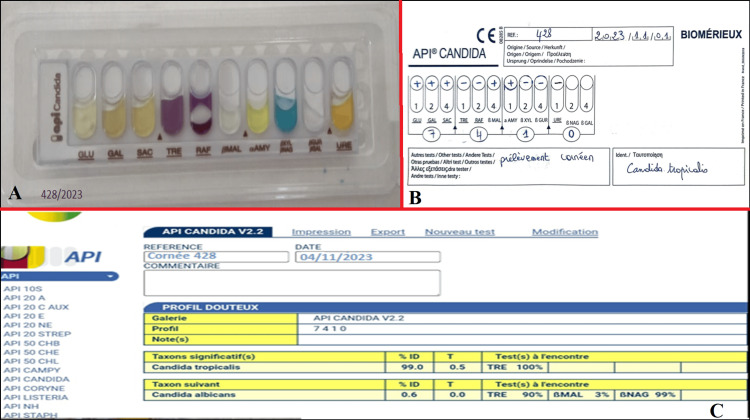
Colorimetric sugar assimilation test system (Api Candida*) (A) with the strain identification code (7410) corresponding to C. tropicalis (B).

**Figure 4:**
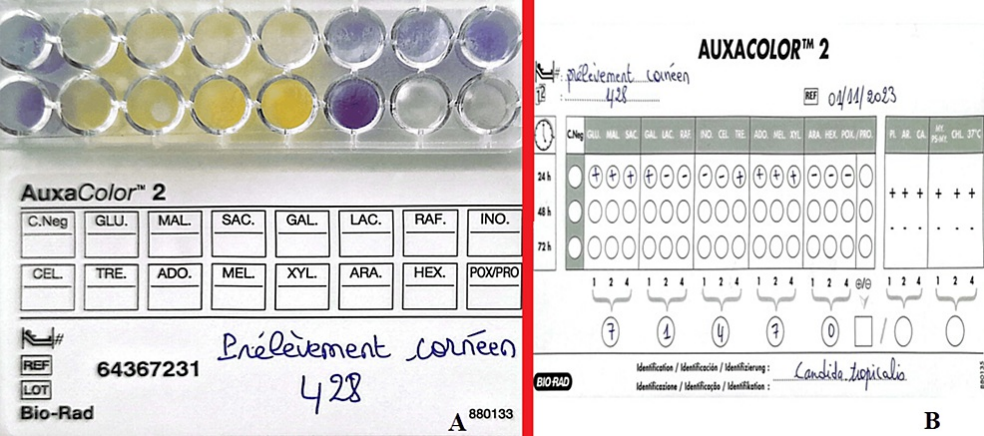
Colorimetric sugar assimilation test system (AUXACOLOR TM 2*) (A) with the strain identification code (71470) corresponding to C. tropicalis (B).

Identification was carried out using the filamentation test, which proved negative, followed by the use of biochemical galleries: an identification system based on sugar assimilation (Api candida 20* [Figure 3] and AUXACOLOR TM 2*: [Figure 4]). After 48 hours of incubation, the code obtained corresponded to *Candida tropicalis*.

**Figure 5:**
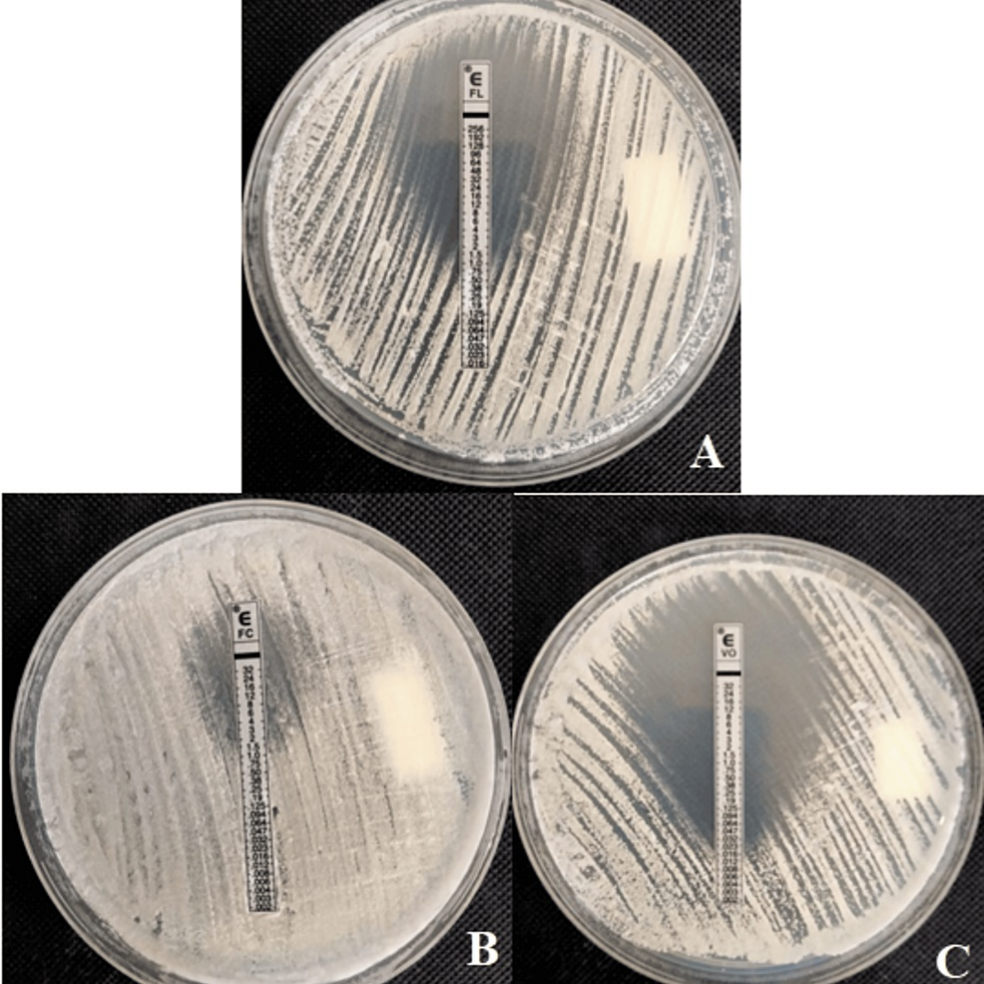
Measurement of minimum inhibitory concentration using the E-test. A) Fluconazole: intermediate (dose-dependent sensitivity), B) Flucytosine: resistant, C) voriconazole: sensitive

Susceptibility to antifungal agents was tested in vitro using the E-test, a technique for assessing fungal susceptibility to three antifungal agents (voriconazole, fluconazole, and flucytosine) by determining the Minimum Inhibitory Concentration (MIC). Results showed sensitivity to voriconazole (Figure 5C), intermediate sensitivity to fluconazole (dose-dependent sensitivity) (Figure 5A), and resistance to flucytosine (Figure 5B).

The patient initially received antibiotic treatment with fortified tobramycin and ciprofloxacin eye drops to treat the superinfection, followed by antifungal treatment in the form of fortified eye drops. The outcome was favorable, with improvements in both the local condition and visual acuity.

The patient was referred for genetic counseling for his corneal dystrophy, and his family members were summoned for an ophthalmologic examination.

## Discussion

Keratomycosis is a leading cause of infectious keratitis globally. It can result in progressive vision loss and potentially spread to other organs [3]. A variety of risk factors are commonly acknowledged, including ocular trauma, corneal surgery, chronic ocular surface disorders, corticosteroid use, and contact lens wear [4]. In this presented case, the patient acquired a corneal infection while having a history of corneal dystrophy, which was the probable trigger for the fungal disease's development.

*Candida* species are the most common yeasts to cause keratitis, particularly in patients whose eyes are already compromised, with *Candida albicans* presenting as the main species [5]. However, over the last few decades, other emerging yeasts of the *Candida* genus have been identified, notably *C. tropicalis*, but its involvement in eye infections has been restricted to isolated cases.

*C. tropicalis* was initially discovered in 1910 by a patient with fungal bronchitis and designated as *Oidium tropicalis* [6]. It is a yeast species classified under the phylum *Ascomycota*, within the class *Ascomycetes* [7]. This yeast is part of the natural human microbiota and is commonly found on the skin, gastrointestinal tract, genitourinary tract, and respiratory system [8]. It has been implicated in both superficial and systemic infections worldwide, particularly in neutropenic patients, in individuals with reduced microbiota due to antimicrobial use, or with lesions of the gastrointestinal mucosa. It can generate true hyphae, a characteristic shared exclusively with *Candida albicans* and its related species, *Candida dubliniensis*. Additionally, *C. tropicalis* is recognized as a proficient producer of biofilms and exhibits strong adherence to epithelial and endothelial cells [9].

*C. tropicalis* is classified as the first or second most common NAC (non-albicans candida) species isolated in clinical practice [10]. The clinical manifestations of *Candida* infections vary based on the affected body area. This genus causes superficial mycoses like oral candidiasis and onychomycosis, whereas systemic candidiasis affects the bloodstream and deep organs, including the lungs and gastrointestinal tract. In the ophthalmological literature, *C. tropicalis* has been described as an emerging cause of endophthalmitis [11,12]. It has been identified as the fourth species of its genus to cause ocular infections in both adult and pediatric patients at two medical centers in the United States of America [13].

In Canada, a seven-year study of microbial keratitis found 34 cases of fungal keratitis, including four cases of yeast, three cases of *Candida albicans*, and a single case of *Candida tropicalis keratitis* [14]. Another 14-year retrospective Canadian study of *Candida keratitis* revealed that of the 21 confirmed cases, *Candida albicans* was the most frequently isolated species, followed by *Candida parapsilosis*. However, *Candida tropicalis* was implicated in two cases [15].

Another study carried out at the Department of Ophthalmology, Bascom Palmer Eye Institute, Miller School of Medicine, University of Miami, involved a comparative analysis of the antifungal susceptibility of corneal isolates of *Candida albicans* versus corneal isolates of *Candida* non-*albicans*. In this study, 68 *Candida* were isolated, including 37 *Candida albicans* and 31 non-*albicans*. *C. tropicalis* accounted for 12.9% of *Candida non-albicans* (four cases), ranking third after *Candida parapsilosis* (19 cases) and *Candida glabrata* (seven cases) [16].

Managing fungal keratitis poses challenges due to the limited and inconsistent sensitivity to antifungal medications, inadequate penetration of topical antifungal agents into tissues, and the potential severity of infections caused by this pathogen. These infections can lead to corneal perforation, endophthalmitis, and irreversible vision loss. However, there are currently no universally accepted standard management protocols for cases of fungal keratitis. The use of antifungal agents within the corneal stroma in cases of keratomycosis has been shown to have an 89% success rate [17].

## Conclusions

Bacterial keratitis is much more common, but fungal keratitis should always be considered, particularly in the context of immunosuppression (corticosteroid use or trauma by a plant). Fungal keratitis caused by *Candida albicans* has long been implicated in corneal infections. Nowadays, new *Candida* species, such as *C. tropicalis*, *C. parapsilosis*, and *C. kruséi*, are being isolated from ophthalmological specimens.

This case of fungal keratitis caused by *Candida tropicalis* highlights the complex challenges of diagnosing and treating this rare but serious corneal infection. The initial presentation and the positive response to antifungal treatment underscore the importance of high clinical suspicion and prompt management to prevent severe complications and improve visual outcomes. Furthermore, this case emphasizes the necessity of multidisciplinary collaboration, including specialists in ophthalmology and microbiology. The key message is the importance of early diagnosis and an appropriate therapeutic approach to optimize recovery chances and preserve vision.

## References

[1] Ortega-Rosales A, Quizhpe-Ocampo Y, Montalvo-Flores M, Burneo-Rosales C, Romero-Ulloa G (2019). A case of fungal keratitis due to Fusarium solani after an indigenous healing practice. IDCases.

[2] Oostra TD, Schoenfield LR, Mauger TF (2018). Candida dubliniensis: A novel cause of fungal keratitis. IDCases.

[3] Shukla PK, Kumar M, Keshava GB (2008). Mycotic keratitis: an overview of diagnosis and therapy. Mycoses.

[4] Erkan Pota Ç, Ayaz Y, Ünal M, Koyuncu Özyurt Ö (2022). Fungal keratitis caused by Scedosporium apiospermum: a case report. J Med Case Rep.

[5] Hassan HM, Papanikolaou T, Mariatos G, Hammad A, Hassan H (2010). Candida albicans keratitis in an immunocompromised patient. Clin Ophthalmol.

[6] 6] Castellani A (1912). Observations on the fungi found in tropical bronchomycosis. Lancet.

[7] 7] Blandin G, Ozier-Kalogeropoulos O, Wincker P (2000). Genomic exploration of the hemiascomycetous yeasts: Candida tropicalis. FEBS Lett.

[8] Negri M, Martins M, Henriques M, Svidzinski TI, Azeredo J, Oliveira R (2010). Examination of potential virulence factors of Candida tropicalis clinical isolates from hospitalized patients. Mycopathologia.

[9] Marcos-Zambrano LJ, Escribano P, Bouza E, Guinea J (2014). Production of biofilm by Candida and non-Candida spp. isolates causing fungemia: comparison of biomass production and metabolic activity and development of cut-off points. Int J Med Microbiol.

[10] Pemán J, Cantón E, Quindós G (2012). Epidemiology, species distribution and in vitro antifungal susceptibility of fungaemia in a Spanish multicentre prospective survey. J Antimicrob Chemother.

[11] Sheu SJ (2017). Endophthalmitis. Korean J Ophthalmol.

[12] Sallam A, Taylor SR, Khan A (2012). Factors determining visual outcome in endogenous Candida endophthalmitis. Retina.

[13] Dozier CC, Tarantola RM, Jiramongkolchai K, Donahue SP (2011). Fungal eye disease at a tertiary care center: the utility of routine inpatient consultation. Ophthalmology.

[14] Harbiyeli İİ, Erdem E, Görkemli N (2022). Clinical and mycological features of fungal Keratitis: a retrospective single-center study (2012-2018). Turk J Ophthalmol.

[15] Qiao GL, Ling J, Wong T, Yeung SN, Iovieno A (2020). Candida Keratitis: epidemiology, management, and clinical outcomes. Cornea.

[16] 16] Spierer O, Dugar J, Miller D, O’Brien TP (2015). Comparative analysis of antifungal sensitivity of corneal isolates of Candida albicans versus non-albicans Candida corneal isolates. Cornea.

[17] Sharma N, Sahay P, Maharana PK (2019). Management algorithm for fungal keratitis: the TST (topical, systemic, and targeted therapy) protocol. Cornea.

